# First report on *Phyllobothrium delphini* infection and *Crassicauda* sp. parasitism resulting in osseous metaplasia in a Cuvier's beaked whale (*Ziphius cavirostris*) from the Brazilian region

**DOI:** 10.1016/j.ijppaw.2021.12.005

**Published:** 2021-12-15

**Authors:** Hassan Jerdy, Max Werneck, Lupercio Barbosa, Rachel Ann Hauser-Davis, Carlos Henrique De-Oliveira-Nogueira, Leonardo Serafim da Silveira

**Affiliations:** a ORCA Institute, Rua José Barcellos de Mattos, 603 B, Parque Areia Preta, Guarapari, ES, Cep 29200-720, Brazil; b Laboratório de Avaliação e Promoção da Saúde Ambiental, Instituto Oswaldo Cruz (Fiocruz), 21040-360, Rio de Janeiro, RJ, Brazil; c Instituto BW, Est. da Praia Seca n, 12.143 (L. 41), CEP 28.970-000, Paia Seca, Araruama, RJ, Brazil

**Keywords:** Bone metaplasia, *Ziphius cavirostris*, *Crassicauda* sp, *Phyllobothrium delphini*, Panniculitis

## Abstract

A female Cuvier's Beaked Whale (*Ziphius cavirostris*) specimen measuring 580 cm in length died after being stranded in Southeastern Brazil. Following a necropsy, organ samples were obtained, fixed in 10% neutral buffered formalin and histopathologically analyzed. A severe and generalized hypodermis infection by *Phyllobothrium delphini* (Phyllobothriidae) was observed, resulting in granulomatous panniculitis. Severe renal and arterial lesions were also noted, including a severe bone metaplasia in the aorta artery, associated with a massive infection by *Crassicauda* sp, (Tetrameridae). A significant thoracic hemorrhage due to thoracic aorta artery rupture was noted, also likely due to this infection, resulting in a fatal injury. This study contributes towards knowledge on histopathologic changes in the scarcely studied Cuvier's Beaked Whale, is the first to associate a *Crassicauda* sp. infection in this whale species in the Brazilian region and also the first to indicate a resulting osseous metaplasia due to this parasitism and granulomatous dermatitis associated with *Phyllobothrium delphini*. Furthermore, this is also, to the best of our knowledge, the first report of *Phyllobothrium delphini* cysts in a *Ziphius cavirostris* specimen to date.

## Highlights

• A new record of *Phyllobothrium delphini* in *Ziphius cavirostris* is reported.
• Detailed *Phyllobothrium delphini* cyst lesions images are provided.
• Rare bone metaplasia is reported for the first time for *Ziphius cavirostris*.
• Bone metaplasia was associated to *Crassicauda* sp. infection.
• This is only the second bone metaplasia report described for cetaceans.

## Graphical abstract

**Figure:**
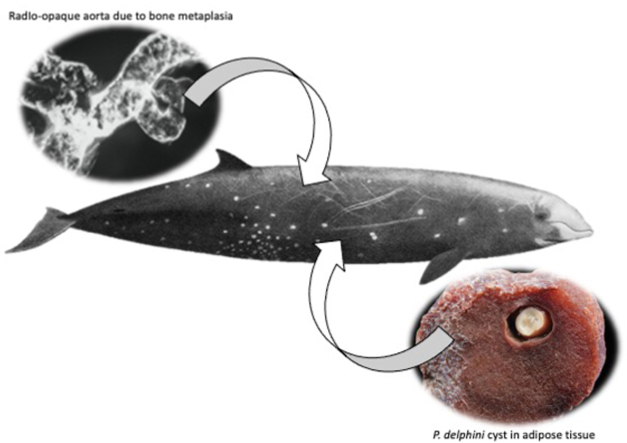

## 1 Introduction

The Ziphiidae family of cetaceans, commonly known as Beaked whales, currently comprise 23 species grouped into six genera, and are one of the least known groups of whales, due to their deep ocean water habits and long diving periods (Bianuci et al., 2014). Parasitological studies, are, in particular, scarce for this group, indicating a knowledge gap that must be filled, as assessing the effects of parasites is essential for designing management and conservation plans of wild animal populations (Balbuena and Simpkin, 2014).

In this regard, current evidence suggests that nematode species belonging to the *Crassicauda* genus, which are exclusively cetacean parasites (Raga and Balbuena, 1990) and known to result in substantial cetacean morbidity and mortality, may play an important regulatory role in cetacean populations (Balbuena and Simpkin, 2014). Infections by this genus have been noted in several cetaceans, such as in the penis of a male long-finned pilot whale (*Globicephala melas*) stranded off the Spanish Mediterranean coast (Raga and Balbuena, 1990), the right heart ventricle and caudal caval vein of a juvenile male fin whale (*Balaenoptera physalus*) in the Channel Terneuzen-Ghent, in Belgium (Lempereur et al., 2017), and in the skulls of pantropical spotted dolphins (*Stenella attenuata*) in the eastern tropical Pacific (Perrin and Powers, 1980). Some publications are available concerning *Crassicauda* sp. infections in Beaked whales, specifically Cuvier's beaked whales (Gomerčić et al., 2006; Díaz-Delgado et al., 2016), and severe lesions, such as cartilaginous metaplasia and calcification in arteries, aneurysms, fibrosing arteritis have been associated to parasitosis events by this genus in these whales (Díaz-Delgado et al., 2016).

Metaplasia is a reversible transformation of adult tissue into another tissue of the same cell germline. Osseous metaplasia is occasionally diagnosed in injured soft tissues (Miller and Zachary 2017), while vascular calcification is defined as mineral deposition in the vasculature, in the form of calcium-phosphate complexes (Lee et al., 2020). Bone metaplasia due to infection by *Crassicauda* sp. however, has not yet been described for cetaceans, even less so associated to other conditions, such as chronic inflammatory processes. Reports on bone metaplasia are, in fact, rare in veterinary medicine, with one report available concerning fish, with no defined etiology (Govett et al., 2004), and two cases associated to chronic inflammation in snakes (Isaza et al., 2000; Nogueira et al., 2016).

In this context, this report aims to further expand information concerning the little-known Cuvier's beaked whale by reporting a parasitosis which causes generalized granulomatous panniculitis and the first case of a disrupted aorta artery, thoracic and abdominal aorta artery with bone metaplasia and renal lesions associated to infection by the parasitic nematode *Crassicauda* sp. This study therefore contributes to knowledge on host-parasite interactions in Cuvier's Beaked whales, which is extremely scarce to date. Furthermore, these new data are of significant value, as *Crassicauda* sp. is a rare example of a parasite whose effects have been quantified in cetacean populations and has been noted as playing an important regulatory role in cetacean populations (Balbuena and Simpkin, 2014).

## 2 Materials and methods

A female Cuvier's beaked whale, 580 cm long, died after being stranded still alive on Itaóca beach (20, 92480 S–40, 79455 W), in the state of Espírito Santo, Brazil, in October 2014. The animal was taken to the Instituto ORCA, a non-governmental organization licensed by the Chico Mendes Institute for Biodiversity Conservation, the Brazilian Ministry of the Environment's administrative arm (ICMBio/MMA), under number 64724. The necropsy lasted about 8 h and was performed according to Geraci and Lounsbury (2005) by three veterinarians with experience in cetacean necropsies and the assistance of a technician. The animal was placed in the prone position, the skin and hypodermis were removed in plates and examined (displaying many live *Phyllobothrium delphini* specimens). After removing excess muscle tissue, the ribs were moved to access and inspect the chest cavity, lung, heart, aorta and esophagus, which were removed and examined. The abdominal cavity was then accessed and examined. Organs from the abdominal cavity were removed and examined individually. Parasites, when found, were collected. Fragments of each tissue were placed in formalin and forwarded to the Department of Morphology and Pathological Anatomy (DMPA) at the Darcy Ribeiro North Fluminense State University Veterinary Hospital (UENF) for histopathological analyses. Samples were fixed in 10% buffered formalin and subsequently cut, dehydrated in an ascending percentage of alcohol solutions, clarified in xylene, embedded by immersion in paraffin, sectioned (5 μm), and stained with hematoxylin-eosin. The slides were then mounted examined under a Nikon Eclipse 80i microscope, using a specific software (NIS Elements).

Parasites found during the necropsy were carefully removed from the whale's tissues using tweezers and brushes, washed in freshwater, and fixed in a 70% alcohol solution. Nematodes were clarified in an Amman lactophenol solution, while parasites encysted in adipose tissue were collected and stained with a hydrochloric carmim solution and clarified with eugenol and others were stained within the cysts. After processing, the parasites were assessed and measured under a Nikon Eclipse 80i microscope through the NIS–Elements–BR software program. The collected helminths were deposited at the Oswaldo Cruz Institute Helminthological Collection (CHIOC- number requested), in the state of Rio de Janeiro, Brazil. Cyst identification in adipose tissue followed Hoberg (1994), Siquier and Le Bas (2003) and Castro et al. (2014), while *Crassicauda* genus identification for followed the identification key proposed by Chabaud (2009) and the revisions by Lambertsen (1985) and Lambertsen (1992).

## 3 Results

The Cuvier's beaked whale carcass was fresh, displaying intact morphology, a 10 cm-thick adipose structure and firm musculature with good muscle tone. A focal recent trauma was associated with mandibular bone exposure at the end of the left mandible in the head region. The specimen exhibited generalized multiple prominent skin lesions with a nodular aspect, occasionally depigmented and centrally ulcerated, diffusely distributed throughout the tegument, which was slightly protruding. The edge of the animal's caudal fin displayed several healed circular perforations measuring about 6 cm in diameter, and the skin displayed miliary nodular lesions diffusely distributed throughout the tegument, and, microscopically, these lesions comprised granulomas that affected the superficial dermis up to the hypodermis. The nodes occasionally exhibited a necrotic center with spongy edges and serosanguineous content at the cut, fibrotic scarring with a fibrous appearance, and white-gray streaks. These nodes presented frequent parasitic cysts. These cysts (0.9 cm wide by 2 cm long) containing parasites in the lesion center were compatible with type A *Phyllobothrium delphini* (Cestodea: Tetraphyllidea) cysts, due to the presence of a “neck” (see Hoberg, 1994; Siquier and Le Bas, 2003).

**Fig. 1:**
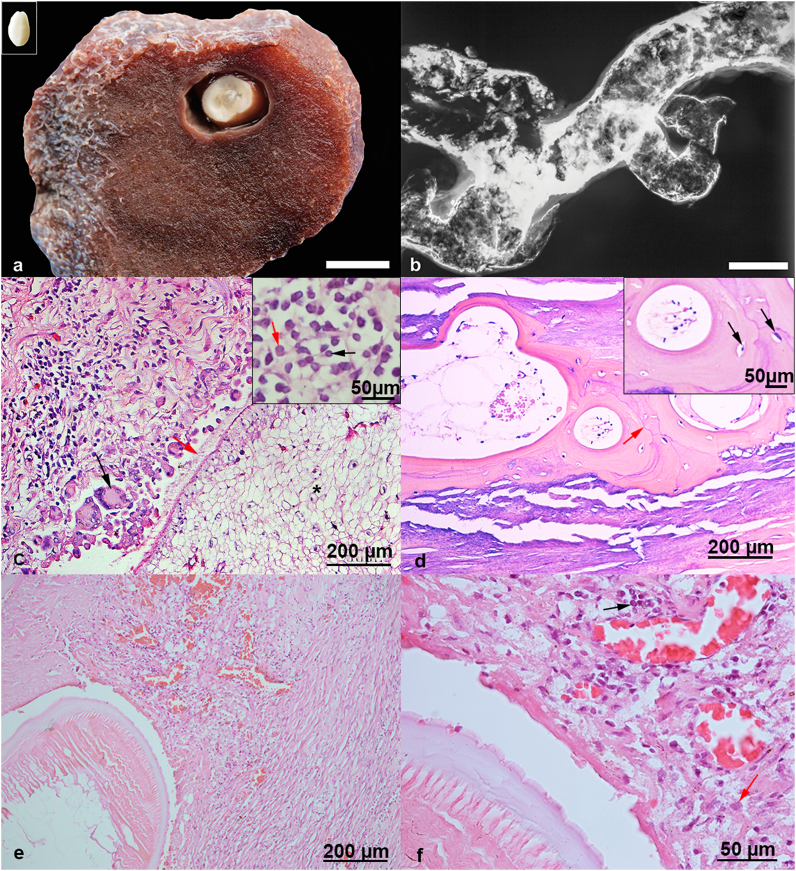
a Infected adipous tissue. *Phyllobothrium delphini* located in the center of the well-delimited parasitic cyst. Inset: *Phyllobothrium delphini* displaying a whitish coloration and shiny appearance. Bar, 2 cm b Thoracic aorta artery displaying bone metaplasia. Radiographic image highlighting the strong radiopacity, similar to bone radiopacity, observed along all the artery walls. Bar, 15 cm c Parasitic cyst located in adipose tissue. Proliferating and reacting fibrous tissue with a moderate number of lymphocytes and plasmocytes located along the parasitic cyst wall. Numerous activated macrophages and multinucleated giant cell (black arrow) are visible around the P. delphini specimen displaying a degenerated cuticle (red arrow). Insert: Hight power field of inflammatory infiltrate with lymphocytes (black arrow) and plasma cells (red arrow). d Thoracic aorta artery displaying bone metaplasia. Mature bone tissue is organized as circumferential lamellae and cement line (red arrow). Note the erythrocytes within the blood vessel and adipose tissue in the medullar region of the detected bone metaplasia. Inset: Hight power field of bone metaplasia with osteocytes allocated in lacunae (black arrow). e Parasitized kidney. *Crassicauda* sp. involved by reactive fibrous tissue associated with inflammatory infiltrate. f Parasitized Kidney. High power field of nephritis associated with *Crassicauda* sp. Note the fibrosis and inflammatory infiltrate formed by macrophages (red arrow) and plasma cells (black arrow). . (For interpretation of the references to color in this figure legend, the reader is referred to the Web version of this article.)

The thoracic cavity of the whale specimen displayed a severe hemothorax (approximately 20 L) and partial thoracic aorta rupture, which was extremely stiff, brittle, sometimes measuring about 1.5 cm in thickness. Aorta stiffening and elasticity loss extended to the abdominal portion of the carcass. Cut surfaces of the thoracic and abdominal aorta had a whitish appearance. The bone metaplasia was very serious, with the aorta so stiff that it looked similar to a plastic PVC pipe. The endothelial surface was deformed and exhibited multiple prominent and rigid cystic formations, yellowish-white with diameters ranging from 0.5 to 1.5 cm. Most of the aorta displayed extensive radiodense areas, compatible with ossification or mineralization upon radiographic examination (Fig. 1). The kidney pelvises, renal calyces, and ureters were dilated and filled with extensive, coiled nematodes. The prominent and dilated renal lobes contained a single coiled parasite inside, pressing on the renal parenchyma. In more severe cases, only a fibrous capsule remained. No parasites were collected whole, although their locations, the collected portions and their dimensions allowed for identification as belonging to the *Crassicauda* genus (Lambertsen, 1985, 1992). When in the renal pelvis, the parasites were entangled with each other. Some parasite fragments measured over 2 m.

Microscopically, a fibrous capsule limited the parasitic cysts in the hypodermis. A massive amount of multinucleated giant Langhans cells and epithelioid macrophages, a moderate number of lymphocytes, plasma cells, and rare eosinophils surrounded the encysted parasites. The parasite cuticle was occasionally dissolved, in contact with the granulomatous inflammatory infiltrate (Fig. 1). A fibrous capsule coated the parasites that died and suffered intense mineralization. The hypodermic tissue adjacent to the parasitic cysts was fibrotic, characterizing parasitic granulomas.

The whale's renal lobes parasitized by *Crassicauda* exhibited a thickened renal capsule due to fibroplasia, extending to the area where the nematodes were lodged, sometimes with marked renal parenchyma atrophy, with practically only the capsule remaining. Reactive connective tissue and a moderate inflammatory infiltrate was observed around the parasite, the first composed of activated macrophages, neutrophils associated with cellular debris, and light mineral deposition compatible with dystrophic mineralization. These findings characterize parasitic pyogranulomatous nephritis.

The tunica intima was absent in several thoracic aorta portions, with few reendothelialization areas. The tunica media of the aorta thoracic and abdominal portion presented a mild inflammatory infiltrate comprising lymphocytes and macrophages with hyperplastic myocytes, characterizing chronic arteritis. Abundant fibroplasia was observed, containing an accentuated number of fibrocytes, rare fibroblasts, and multifocal to coalescent foci of eosinophilic tissue with cells allocated in lacunae that formed osteoid, mature bone tissue (Fig. 1). In some portions, the intima and tunica media were completely replaced by reactive fibrous tissue, bone tissue, and mineralization. The formation of tissue composed of cells accommodated in lacunae compatible with a basophilic chondroid matrix was rarely noted. These findings are characteristic of bone metaplasia and cartilaginous metaplasia. Viable nematode eggs were found in some histological sections of the renal veins, and the arterial lumen displayed nematode larvae, characterizing parasitic embolism.

Furthermore, the cytoplasm of the Cuvier's beaked whale neurons exhibited moderate to significant amounts of lipofuscin in the central nervous system (spinal cord, cerebellum, and brain).

## 4 Discussion

The findings reported herein of endothelium loss, scarce reendothelialization, arterial thickening, hyperplastic myocytes, reactive fibrous tissue in the intima and tunica media, cartilaginous metaplasia, inflammatory infiltrate composed of lymphocytes and macrophages, larvae in the lumen of blood vessels have been previously described in another Cuvier's Beaked whale specimen (Díaz-Delgado et al., 2016). Furthermore, the renal lesions observed in our Cuvier's Beaked whale specimen, such as dystrophic mineralization, pyogranulomatous nephritis associated with necrosis, and renal fibrosis, were also similar to those reported by Díaz-Delgado et al. (2016). However, in addition to these findings, free nematode eggs in renal veins were also observed in our whale specimen. Bone metaplasia, though, has never been described in association with parasitic infection in cetaceans. In fact, only one description of bone metaplasia is available for this group, associated, however, with severe skin lesions in a dolphin found entangled in fisher nets (St. Leger et al., 2018). Bone metaplasia in mammals is mainly caused by tumors (Kornegay et al., 1991; Sant'Ana et al., 2002; Bangari and Stevenson 2007; Kannegieter et al., 2010; Ramirez et al., 2010; Ohfuji 2012), followed by metabolic and eating disorders (Han and Gardiner, 2016).

Changes regarding vascular wall thickening and distortion, calcification, and occasional cartilage metaplasia can result in severe lesions such as fibrosing arteritis, aneurysms, hemorrhages, and thrombosis, involving the vascular system in Cuvier's beaked whales (Díaz-Delgado et al., 2016). In this case report, the animal presented a severe thoracic hemorrhage associated with thoracic aorta rupture, indicating that it may have died as a result of hypovolemic shock.

The high amount of lipofuscin observed in the cytoplasm of the specimen's neurons is associated with cellular aging, as, the more lipofuscin present, the older the cell (Kumar et al., 2015). Therefore, as our findings indicated a moderate to high amount of lipofuscin in neurons, the whale was determined as of an advanced age.

*P. delphini* cysts are commonly found in the adipose tissue of cetaceans and have been described in several species (see Siquier and Le Bas, 2003 and references therein). In Brazil, this parasite has been previously described for the following cetaceans: Rough-toothed dolphin (*Steno bredanensis)*, humpback whale (*Megaptera novaeangliae)*, sperm whale (*Physeter macrocephalus)*, dwarf sperm whale (*Kogia sima)*, pygmy sperm whale (*Kogia breviceps)*, melon-headed whale (*Peponocephala electra)*, Clymene dolphin (*Stenella clymene)*, striped dolphins (*Stenella coeruleoalba)*, Atlantic spotted dolphin (*Stenella frontalis)*, spinner dolphins (*Stenella longirostris)*, and Fraser's dolphin (*Lagenodelphis hosei)* (Luque et al., 2010). Our report is, however, to the best of our knowledge, the first report of *P. delphini* cysts in *Ziphius cavirostris*, and no macroscopic or histopathological descriptions of *P. delphini* infection for this whale species are available in the specialized literature.

*Crassicauda* sp. infections in cetaceans, on the other hand, are well documented (Marcer et al., 2019), including reports of arterial lesions in a *Z. cavirostris* specimen from the Canary Islands (Díaz-delgado et al., 2016). This report, however, comprises the first report for this parasitism in the Brazilian region.

Parasitic and bacterial infections may occur due to many factors, such as environmental chemical contamination, habitat degradation and climate change effects, among others (Siciliano et al., 2020) and can drive immune and inflammatory responses, causing chronic tissue damage and resulting in ectopic ossification (Demer and Tintut, 2014). Calcification of the tunica media favors remodeling and mineralization episodes, with a consequent decrease in vascular elasticity (Niskanen et al., 1994). In the present study, the aorta was brittle and lacked elasticity, consequently losing resistance, a factor that contributed to its rupture and the subsequent thoracic hemorrhage. Microscopically, the observed bone metaplasia was considered a consequence of chronic arterial inflammation associated with the presence *Crassicauda* sp. larvae.

## 5 Conclusions

A massive infection by *Crassicauda* sp. nematodes resulted in serious cardiovascular and urinary system disorders in a Cuvier's Beaked whale specimen found stranded alive in the state of Espírito Santo, Brazil. Furthermore, this infection was indirectly associated with a severe aorta rupture and thoracic aorta artery metaplasia, leading to an acute hemothorax in the elderly whale specimen, which was the only observed finding that could lead to the animal's sudden death. The macroscopic and microscopic descriptions for the observed *Phyllobothrium delphini* infection are the first for this whale species, resulting in parasitic granulomatous panniculitis. This study contributes towards better insights of the effects of parasitic infections in cetacean species, comprising the first report for a Cuvier's Beaked whale specimen *Crassicauda* sp. infection in the Brazilian region, and the first to associate this parasitism condition to bone metaplasia.

## Financial support

This research received no specific grant from any funding agency, commercial or not-for-profit sectors.

## 6 Statement of interest

None.

## Ethical standards

No experimentation was performed in this studyc

## Declaration of competing interest

None.
